# The Effect of Training with Weightlifting Catching or Pulling Derivatives on Squat Jump and Countermovement Jump Force–Time Adaptations

**DOI:** 10.3390/jfmk5020028

**Published:** 2020-05-01

**Authors:** Timothy J. Suchomel, Shana M. McKeever, John J. McMahon, Paul Comfort

**Affiliations:** 1 Department of Human Movement Sciences, Carroll University, Waukesha, WI 53186, USA; smckeeve@carrollu.edu; 2 Directorate of Sport, Exercise, and Physiotherapy, University of Salford, Salford, Greater Manchester M6 6PU, UK; J.J.McMahon@salford.ac.uk (J.J.M.); p.comfort@salford.ac.uk (P.C.); 3 Centre for Exercise and Sports Science Research, Edith Cowan University, Joondalup, WA 6027, Australia

**Keywords:** vertical jump, impulse, time normalization, strength, power

## Abstract

The purpose of this study was to examine the changes in squat jump (SJ) and countermovement jump (CMJ) force–time curve characteristics following 10 weeks of training with either load-matched weightlifting catching (CATCH) or pulling derivatives (PULL) or pulling derivatives that included force- and velocity-specific loading (OL). Twenty-five resistance-trained men were randomly assigned to the CATCH, PULL, or OL groups. Participants completed a 10 week, group-specific training program. SJ and CMJ height, propulsion mean force, and propulsion time were compared at baseline and after 3, 7, and 10 weeks. In addition, time-normalized SJ and CMJ force–time curves were compared between baseline and after 10 weeks. No between-group differences were present for any of the examined variables, and only trivial to small changes existed within each group. The greatest improvements in SJ and CMJ height were produced by the OL and PULL groups, respectively, while only trivial changes were present for the CATCH group. These changes were underpinned by greater propulsion forces and reduced propulsion times. The OL group displayed significantly greater relative force during the SJ and CMJ compared to the PULL and CATCH groups, respectively. Training with weightlifting pulling derivatives may produce greater vertical jump adaptations compared to training with catching derivatives.

## 1. Introduction

Vertical jumping may be one of the most common movements in sports and is regularly assessed within an athlete monitoring program [1]. The most commonly assessed vertical jump variations include the squat jump (SJ), countermovement jump (CMJ), and drop/depth jump. While jump height is a commonly assessed variable, some researchers have suggested that jump height alone is limited in its capacity to assess an athlete’s performance [2,3]. Because jump height is determined by relative net impulse [4], researchers have suggested examining jump height with its mechanistic underpinnings of force and time during different phases of a jump [1,5,6]. The rationale behind this is that the same jump height could theoretically be achieved with high forces over a short duration or lower forces over a longer duration. Thus, by examining jump height and its underpinning variables together, sport science practitioners can evaluate the strategy used to achieve a specific jump height. Given that vertical jumps, as well as other movements in sports, are often performed with time constraints, rapid force production during the propulsion phase of a movement is crucial. Thus, it is important to regularly assess such characteristics as well as to implement training strategies that may benefit rapid force production. 

There are several different resistance training methods that have been used to improve vertical jump performance (e.g., powerlifting, weightlifting, plyometric training, kettlebells, etc.). The results of a previous meta-analysis indicated that training with weightlifting movements (e.g., clean, snatch, and their variations) may benefit vertical jump performance in athletic populations [7]. In fact, researchers have shown that incorporating weightlifting exercises may produce greater adaptations compared to those obtained by other training methods [8,9,10]. Such improvements are likely due to the similarities between the coordinated triple extension of the hip, knee, and ankle joints (plantar flexion) during jumping and the second pull movement of weightlifting exercises [11,12]. In addition, weightlifting movements allow individuals to provide a greater force overload stimulus that may be limited when using other training methods [10,13,14]. 

Although weightlifting movements are commonly implemented in resistance training programs [15,16], they are often exclusively prescribed with the catch phase. Despite the many benefits of weightlifting catching derivatives, weightlifting pulling derivatives (i.e., exercises that remove the catch phase) have been shown to provide a comparable [17,18,19] or greater [17,18,19,20,21,22,23,24,25] strength–power training stimulus. Despite the existence of previous cross-sectional research, limited training study information is present. Comfort et al. [26] indicated that training with load-matched weightlifting catching or pulling derivatives for eight weeks produced similar improvements in SJ, CMJ, and isometric mid-thigh pull performance. While these findings provide some insight on the training effects of load-matched weightlifting catching and pulling derivatives, it should be noted that pulling derivatives may provide a greater force and velocity overload stimulus compared to catching derivatives via the appropriate manipulation of both load and exercise [14,27]. Specifically, pulling derivatives may be prescribed with loads in excess of a one repetition maximum (1RM) catching variation (e.g., mid-thigh pull and countermovement shrug [28,29,30,31,32]) to benefit force production (i.e., strength) or submaximal loads with more ballistic exercises (e.g., jump shrug and hang high pull [21]) to benefit velocity characteristics. A recent study compared the training effects of load-matched catching or pulling derivatives and weightlifting pulling derivatives that included a force and velocity overload stimulus [33]. The results of the previous study showed that training with pulling derivatives that use a force and velocity overload stimulus resulted in the greatest improvements in multi-joint isometric and dynamic strength, short sprint, and change of direction performance. However, no research has examined or compared the training effects of this type of stimulus on vertical jump performance. Therefore, the purpose of this study was to compare the SJ and CMJ training adaptations following a training program that features either weightlifting catching (CATCH), pulling derivatives (PULL), or pulling derivatives that include a force and velocity overload stimulus (OL). It was hypothesized that the OL group would produce the greatest improvements in SJ and CMJ force–time characteristics, while no differences will exist between the CATCH and PULL groups in line with previous research [26]. The authors would like to acknowledge that the data from the present study and a previous study [33] are related and were collected as part of the same project. However, the authors felt that the vertical jump data needed to be presented separately in order to provide a more thorough analysis of SJ and CMJ force–time (continuous) data (compared to the discrete data in the previous study), while also respecting word, figure, table, and reference limits of the previous journal and not overwhelming readers with large datasets. 

## 2. Materials and Methods 

### 2.1. Participants

Twenty-seven male collegiate athletes and resistance-trained men with previous experience with the power clean (PC) and its derivatives volunteered to participate in this study. Each participant was assigned to either the CATCH, PULL, or OL group (Table 1) using a covariate-based randomization process [34]. Two participants were excluded from the analysis because their jump data were identified as outliers. Training session attendance for those that completed the study was 100%. Each participant read and signed a written informed consent form prior to their participation in the study, in accordance with Carroll University’s institutional review board (#17-017, approved 10 May 2017). 

An a priori power analysis was completed using G*Power (version 3.1.9.2). Based on previous findings [35], it was determined that ≥24 participants were required to display at least moderate effect sizes (Hedge’s *g* ≥ 0.55) between groups at a power level of 0.90 and an a priori alpha level of ≤0.05.

### 2.2. Procedures and Design

**Figure 1:**
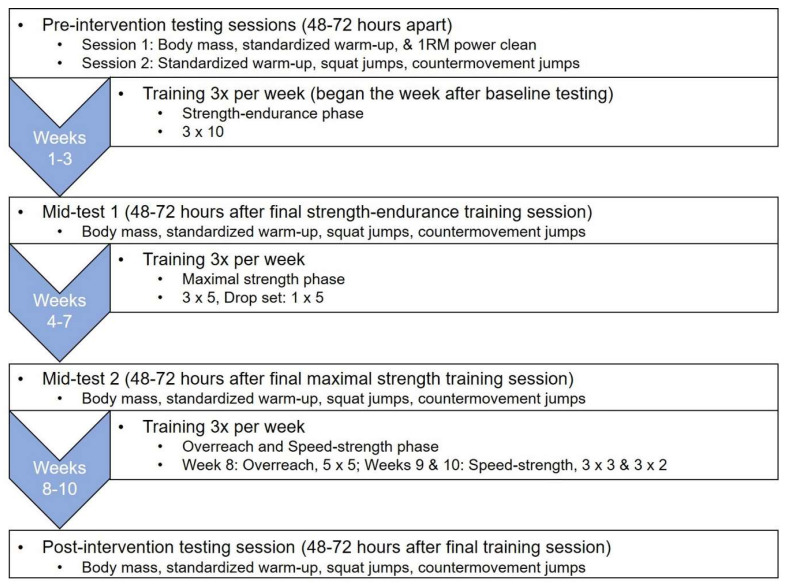
Testing and training sequence. Sets × repetitions.

A repeated-measures within- and between-group design was used to examine the differences in SJ and CMJ performance and underpinning force–time curve characteristics following resistance training programs that used weightlifting catching or pulling derivatives. The participants completed 10 weeks of training (three times per week) and were assessed prior to the training intervention and again after 3, 7, and 10 weeks of training (Figure 1). Each testing session was scheduled to take place within two hours of the participants’ pre-intervention testing sessions to ensure that testing took place during a comparable time of day, to minimize the time-of-day effect. Prior to each testing session, the participants performed the same standardized warm-up that consisted of stationary cycling, dynamic stretching, body weight squats, and progressive vertical jumps [20,23,36].

### 2.3. 1RM Power Clean Assessment

Participants arrived at the performance laboratory in a hydrated state having refrained from strenuous exercise for at least 48 h for their 1RM PC testing session. After the participant was weighed, they completed the aforementioned standardized warm-up. Following the warm-up, the participants performed a self-selected warm-up with a 20 kg barbell before completing warm-up PC sets using submaximal loads (five repetitions at 30% and 50%, three repetitions at 70%, and one repetition at 90% of their estimated 1RM), in accordance with previous procedures [37]. Following the final warm-up set, the principal investigator and the participant determined the first maximal attempt load. A minimum 2.5 kg increase was required, and loads were progressively increased until the participant recorded a failed attempt. Participants were given at least three minutes of rest in between 1RM attempts. Any PC caught with the top of the subject’s thigh below parallel (visually monitored) was ruled an unsuccessful attempt. 

### 2.4. Vertical Jump Assessment

All SJ and CMJ trials were performed with the participants standing on dual force plates (PASPORT, PASCO Scientific, CA, USA) sampling at 1000 Hz while holding a polyvinyl chloride (PVC) pipe (<1 kg) across their upper back. Before the maximal effort trials of each testing session, each participant was familiarized/re-familiarized with the SJ “ready position” by squatting to a knee angle of approximately 90°. The principal investigator then verified the knee angle twice using a manual goniometer. Next, each participant performed warm-up SJs at 50% and 75% of their perceived maximum effort. During the maximal trials, the participants were instructed to squat to the same ready position, briefly hold this position, receive a countdown of “3, 2, 1, Jump!”, and jump as high as possible. All SJs were performed without a countermovement using a concentric-only motion. If a countermovement was displayed within the force–time record of an SJ trial, based on visual inspection, another trial was performed after a one minute rest period. Following the SJ trials, each participant performed maximal CMJ trials. Prior to the maximal effort trials, each participant performed warm-up CMJs at 50% and 75% of their perceived maximum effort. When performing the CMJs, the participants received the same countdown of “3, 2, 1, Jump!” After the countdown, the participants performed a countermovement to a self-selected squat depth and jumped as high as possible. Two maximal effort jumps, with one minute of rest between jumps, were performed for both the SJ and CMJ conditions, and the average performance of both jumps was used for statistical analysis.

### 2.5. Training Intervention

The CATCH, PULL, and OL groups each trained three days per week for 10 weeks under the supervision of a certified strength and conditioning coach (Table 2). The resistance training program was modified from a recent review article [27]. Similar to previous research [21,26,29,38,39], the loads for each weightlifting catching and pulling derivative were programmed based on a 1RM PC achieved during a pre-intervention testing session. Each weightlifting derivative was coached using the technique described in previous literature [37,40,41,42,43,44]. Because weightlifting derivatives are rarely programmed in isolation, non-weightlifting derivative exercises were added to the training intervention to increase the ecological validity of each program. The set-repetition best method [45,46,47] was used to estimate the 1RMs for the non-weightlifting derivative exercises (e.g., back squat, bench press, bent-over row, etc.) using the participants’ recent training history. It should be noted that the 1RMs for each non-weightlifting exercise were readjusted following week 7 of the training program. The relative loads prescribed for each training phase are displayed in Table 3. Using this method of loading, relative loads were based on percentages of the RM of the prescribed repetitions. For example, 95% of three sets of five repetitions uses 95% of the participant’s estimated 5RM weight. However, a range of loads was prescribed to allow participants to determine the appropriate loads based on how many repetitions they feel that they could have performed beyond the prescribed number of repetitions [46]. Relative intensities were described as very light (65–70%), light (70–75%), moderately light (75–80%), moderate (80–85%), moderately heavy (85–90%), heavy (90–95%), and very heavy (100%) [45]. Finally, it should be noted that when performing three sets of 10 repetitions, weightlifting derivatives were prescribed using cluster sets of 5 repetitions with 30–40 s of intra-set rest [48].

The differences between each of the training programs are displayed in Table 3. The CATCH group trained using PC derivatives that included the catch phase during every repetition, while the PULL and OL groups trained using biomechanically similar PC derivatives that excluded the catch phase. The volume-load between the CATCH and PULL groups was matched, where each group performed their derivatives with the same relative loads based on their 1RM PC (e.g., CATCH = mid-thigh PC at 55% 1RM; PULL = mid-thigh pull at 55% 1RM). In contrast, the OL group performed their PC derivatives with either a force or velocity overload stimulus, using either heavier (e.g., CATCH = mid-thigh PC at 55% 1RM; OL = mid-thigh pull 110% 1RM) or lighter loads (e.g., CATCH = hang PC at 60% 1RM; OL = jump shrug at 35% 1RM), respectively. Weightlifting pulling derivatives that are more ballistic in nature (e.g., jump shrug) were also used to provide the velocity overload stimulus [20,21,23]. Although the volume-load was different between the OL group and the other groups, this method of loading was meant to increase the ecological validity of prescribing pulling derivatives based on previous recommendations [27]. 

### 2.6. Data Analysis

The raw force–time data from each SJ and CMJ trial were collected directly from the force plates using a laptop computer and specialized software (PASCO Capstone, PASCO Scientific, CA, USA). The unfiltered data [49] were then exported to and graphed in a customized spreadsheet (Microsoft Inc., Redmond, WA, USA). Each participant stood motionless with the PVC pipe on the force plates prior to each jump to determine the system weight (body mass + PVC pipe) [49]. The onset threshold of each jump was then determined by taking the standard deviation of the vertical ground reaction force across the first second and multiplying it by five. Based on previous recommendations [49], the onset of each jump was considered to have occurred 30 ms before the instant where vertical force increased above or decreased below the calculated threshold with respect to system weight. Force–time data were integrated to generate the velocity–time curves. Using the velocity–time data, the power–time curves of each trial were determined using the product of the given force and velocity data at each time point. The velocity–time record was also used to determine the onset of the propulsive phase for each CMJ trial by identifying the instant where velocity first exceeded 0.01 ms^−1^ following the onset of the jump. The performance variables for both the SJ and CMJ included jump height, propulsion mean force, and propulsion time. Jump height was determined using the velocity at take-off method [50], in which take-off was identified as the instant where force was less than five times the standard deviation of the residual force [49]. Propulsion mean force was calculated as the average force produced during the propulsion period, which started after the system center of mass velocity exceeded 0.01 ms^−1^ and ended at take-off (as identified above). Relative mean force was then calculated by dividing the propulsion mean force by the participant’s body mass. Propulsion time was calculated as the time between the onset propulsion phase of each jump (as determined by a center of mass velocity that exceeded 0.01 ms^−1^) to take-off. The force–time curves were time-normalized by equalizing the number of samples contained in each curve by adjusting the time delta between samples and then resampling the signal [23]. This was done by using a customized spreadsheet (Microsoft Excel, Microsoft Inc., Redmond, WA, USA) to calculate a time normalization factor by dividing the number of samples from the onset of movement (as identified above for the SJ and using less than five times the standard deviation of 1 s of the quiet standing force before the unweighting phase of the CMJ [49]) to take-off by 500. Using this equation, force data were resampled to 500 data points, where each data point corresponded to 0.2% of the total movement time. An offset factor for each data point was then calculated as the product of the resample number (i.e., 0–500) and time normalization factor. From here, the OFFSET function was used to reference the raw force–time data and return the force value that corresponded to the previously calculated offset factor (e.g., an offset factor of 10 returned the 10th force sample from the onset of movement). While all of the raw force–time data were collected at 1000 Hz for the vertical jumps, as mentioned above, the time normalization resampling process required the sampling frequency to be adjusted for each participant based on their unique movement times during SJ (967–1502 Hz) and CMJ (533–933 Hz) trials. The force–time curves were expressed as a percentage (0–100% of the jump) for comparison purposes. The average of each variable and time-normalized force–time curve was used for statistical analysis. 

### 2.7. Statistical Analyses

The distribution of all the data was examined using the Shapiro–Wilk test of normality. Outliers were identified and removed if the data point was greater than three times the standard deviation of the training group’s mean. Levene’s test was used to assess the heterogeneity of variance between groups. Relative and absolute reliability for each variable was assessed during each testing session using two-way mixed intraclass correlation coefficients (ICC) and typical error expressed as a coefficient of variation percentage (CV%). The ICCs were interpreted as poor (<0.50), moderate (0.50–0.74), good (0.75–0.90), and excellent (>0.90) [51]. Acceptable within-session variability was classified as <10% [52]. A series of 3 (group) × 4 (time) mixed ANOVA with Bonferroni post hoc analyses were used to examine the changes within and between the CATCH, PULL, and OL groups. If the assumption of sphericity was violated, Greenhouse–Geisser adjusted *p*-values were reported. A criterion *p*-value of ≤0.05 was used to identify statistical significance. In addition, the magnitude of any changes were determined by calculating Hedge’s *g* effect sizes with a correction for small sample bias [53] (Equation (1)). Effect sizes were interpreted as trivial, small, moderate, and large when magnitudes were <0.25, 0.25–0.49, 0.50–1.0, and >1.0, respectively, based on the “highly trained” (i.e., individuals training for at least 5 years) scale noted within previous literature [54]. Pre- and post-intervention force–time curves were compared using 95% confidence intervals calculated at each time point with the time-normalized curves for each training group. All statistical analyses were performed using SPSS (Version 26, IBM, New York, NY, USA).

(1) $$Hedge^{\prime }s\;g=\frac{M_{2}-M_{1}}{SD^{*}pooled}\cdot \left(1-\frac{3}{4\left(n_{1}+n_{2}-2\right)-1}\right)\;$$

Equation (1) Hedge’s *g* with bias correction. *M_2_ − M_1_* = difference in means; SD*pooled = pooled and weight standard deviation; n_1_ + n_2_ = sum of the sample sizes corresponding to each mean.

## 3. Results

All data were normally distributed and there were no statistically significant differences in variance between groups (*p* > 0.05). In addition, all variables displayed good–excellent within-session reliability and acceptable variability (Table 4).

### 3.1. Between- and Within-Group Comparisons

**Figure 2:**
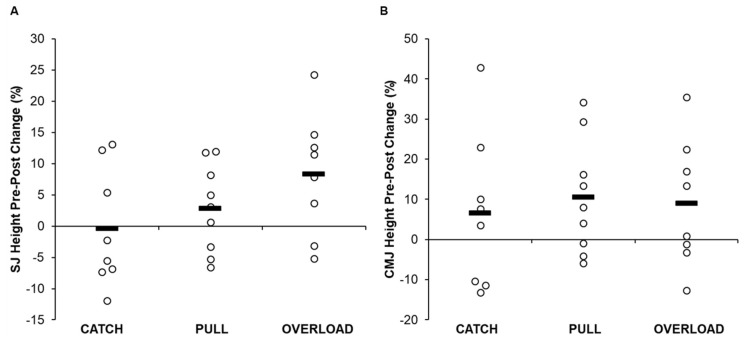
Pre- and post-intervention individual changes in squat jump (**A**) and countermovement jump (**B**) height. Bold line denotes group average and open circles denote individual changes.

**Figure 3:**
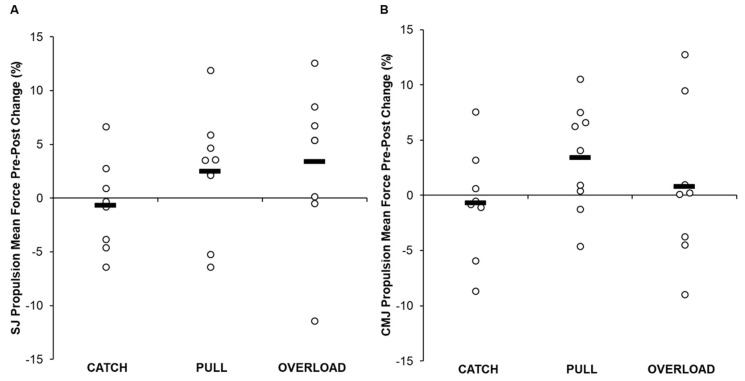
Pre- and post-intervention individual changes in squat jump (**A**) and countermovement jump (**B**) propulsion mean force. Bold line denotes group average and open circles denote individual changes.

**Figure 4:**
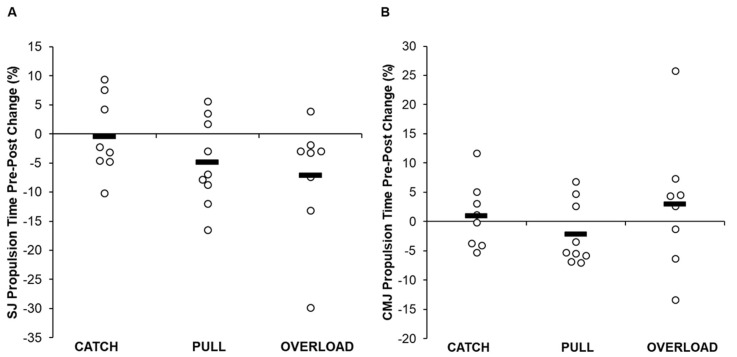
Pre- and post-intervention individual changes in squat jump (**A**) and countermovement jump (**B**) propulsion time. Bold line denotes group average and open circles denote individual changes.

Descriptive data for each group are displayed in Table 5 and Supplementary Materials, and individual changes within each group are displayed in Figure 2, Figure 3 and Figure 4. A statistically significant group × time interaction was present for SJ propulsion mean force (*p* = 0.039); however, post hoc analysis revealed that no differences existed between groups (*p* = 0.943–1.000). In addition, significant differences were present for SJ propulsion mean force across time points for the PULL group (*p* = 0.007); however, post hoc analysis revealed no differences between testing sessions. No other statistically significant interaction effects were present for SJ height (*p* = 0.298) or propulsion time (*p* = 0.059), or for CMJ height (*p* = 0.814), propulsion mean force (*p* = 0.606), or propulsion time (*p* = 0.652).

There were no statistically significant between-group differences for SJ height (*p* = 0.515), propulsion mean force (*p* = 0.589), or propulsion time (*p* = 0.338) or for CMJ height (*p* = 0.550), propulsion mean force (*p* = 0.333), or propulsion time (*p* = 0.428). 

Finally, statistically significant within-group differences in SJ propulsion time across time points existed for the CATCH (*p* = 0.006) and PULL (*p* = 0.017) groups. It should be noted that the OL group also reached significance for SJ propulsion time; however, because the assumption of sphericity was violated, the Greenhouse–Geisser adjusted value did not meet the significance criteria. Post hoc analyses are displayed in Table 5. Specifically, SJ propulsion time was greater during mid-intervention test 1 and post-intervention compared to that during mid-intervention test 2 for the CATCH and PULL groups, respectively. There were no other within-group differences across testing sessions for any group (*p* > 0.05).

### 3.2. Force–Time Curve Comparisons

**Figure 5:**
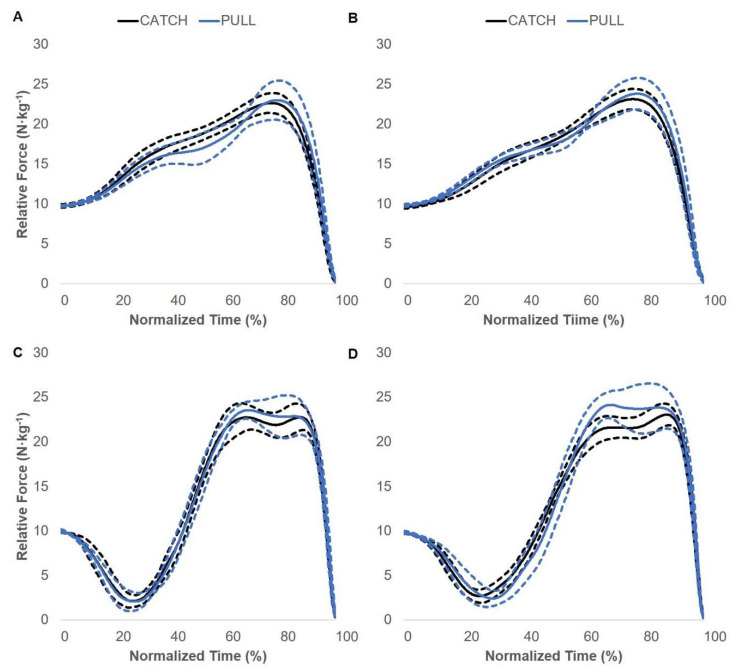
CATCH and PULL group squat jump and countermovement jump force–time curve adaptations. Pre- (**A**) and post-intervention (**B**) squat jump force–time curve adaptations. Pre- (**C**) and post-intervention (**D**) countermovement jump force–time curve adaptations. Solid and dashed lines display the group average and 95% confidence intervals, respectively.

**Figure 6:**
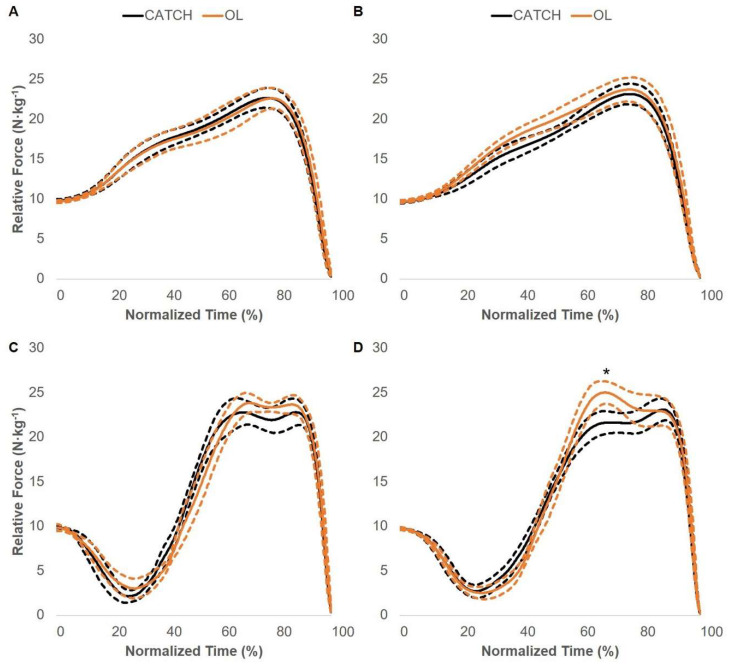
CATCH and OL group squat jump and countermovement jump force–time curve adaptations. Pre- (**A**) and post-intervention (**B**) squat jump force–time curve adaptations. Pre- (**C**) and post-intervention (**D**) countermovement jump force–time curve adaptations. Solid and dashed lines display the group average and 95% confidence intervals, respectively. * = statistically significant difference in relative force.

**Figure 7:**
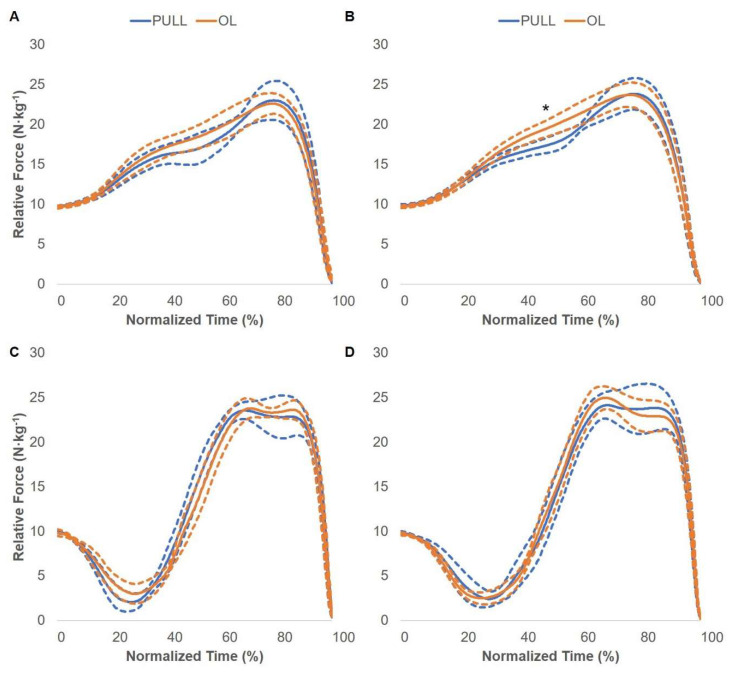
PULL and OL group squat jump and countermovement jump force–time curve adaptations. Pre- (**A**) and post-intervention (**B**) squat jump force–time curve adaptations. Pre- (**C**) and post-intervention (**D**) countermovement jump force–time curve adaptations. Solid and dashed lines display the group average and 95% confidence intervals, respectively. * = statistically significant difference in relative force.

Pre- and post-intervention SJ and CMJ between-group force–time curve comparisons are displayed in Figure 5, Figure 6 and Figure 7. There were no statistically significant differences between the CATCH and PULL groups. The OL group displayed statistically greater relative force compared to the CATCH group during the post-intervention CMJ comparison. Specifically, the OL group produced greater relative force at 63–74% of the total movement time. In addition, the OL group displayed statistically greater relative force compared to the PULL group during the post-intervention SJ comparison. Specifically, the OL group produced greater relative force at 41–56% of the total movement time. No other differences were present.

## 4. Discussion

The current study compared the effects of training with either load-matched weightlifting catching or pulling derivatives or with a force- and velocity-specific overload stimulus using pulling derivatives. The current findings show that there were no statistically significant differences between groups for any of the examined variables. In addition, none of the examined groups displayed statistically significant differences from pre- to post-intervention in any of the examined variables, although several small–moderate effect sizes were present for the PULL and OL groups. In contrast, trivial changes existed for the CATCH group. Finally, the OL group displayed significantly greater relative force production within the SJ and CMJ force–time curves post-intervention compared to the CATCH and PULL groups, respectively.

The results of the current study show that although there were no significant differences between groups or within each group, the greatest improvements in SJ height were shown by the OL group. Given that jump height is determined by the relative net propulsive impulse [4], we sought to examine variables directly affecting jump height. Regarding the SJ, both the PULL and OL groups increased mean propulsion force (2.5% and 3.4%) and decreased propulsion time (−4.9% and −7.2%). In contrast, the CATCH group displayed no significant or practically meaningful changes in SJ performance. These findings contradict the previous study by Comfort et al. [26], who showed increases in SJ height in both load-matched CATCH and PULL groups with no meaningful difference between them. Interestingly, force–time curve analysis displayed a statistically significant difference during 15% of the total movement time favoring the OL group. Therefore, by combining greater propulsive mean forces over a shorter duration, it appears that the OL group displayed the most practically significant changes in SJ performance. These findings may be explained by the inclusion of several weightlifting pulling derivatives that used loads in excess of their 1RM PC (e.g., mid-thigh pull, countermovement shrug, and clean pull from the floor) within the training program of the OL group. Previous literature suggests that using pulling derivatives in this manner may provide a greater force overload stimulus compared to training with submaximal loads [14,27,33]. Moving heavier loads from a static position with maximal intent mimics the demands of the SJ, thus allowing for greater transfer to performance.

Each of the examined training groups, on average, increased their CMJ performance. The greatest improvements in CMJ height were realized by the PULL group (10.5%). The PULL group was followed in order by the OL (9.0%) and CATCH (6.5%) groups. The improvements in CMJ height of the PULL group were underpinned by increases in mean propulsion forces (3.4%), while propulsion time remained relatively unchanged. The differences in performance increases between the CATCH and PULL groups are again in contrast with Comfort et al. [26], who showed improvements in CMJ height in both load-matched CATCH and PULL groups, but no significant or practically meaningful differences between groups. The training program of the PULL group was highlighted by using weightlifting pulling derivatives with the same loads that the CATCH group used. Previous literature has indicated that weightlifting pulling derivatives may produce similar [17,18,19] or greater [17,18,19,20,21,22,23,24,25] force production characteristics (e.g., peak force, velocity, power, etc.) when performed at the same loads as catching derivatives. Because several weightlifting pulling derivatives may use loads in excess of a 1RM catching variation [28,29,30,31,32], it could be argued that the theoretical 1RM of some pulling derivatives may be much greater. Thus, the current training program for the PULL group allowed the participants to train using theoretically submaximal loads with maximal intent and may have contributed to their improvements in propulsive mean force, but also allowed the participants to move rapidly through the stretch-shortening cycle action that occurs during the transition phase of weightlifting movements [55]. However, further analysis should be completed before drawing this conclusion. 

Although not a primary focus in the current study, the pre- to post-intervention force–time curves displayed changes to the peak braking (eccentric) force production characteristics during the CMJ [56] that were unique to each group (CATCH = −5.0%, PULL = 2.5%, OL = 5.0%). Although one of the benefits of weightlifting catching derivatives is the ability to train braking forces when accepting an external load in the rack position [57], the findings of the current study indicate that the braking characteristics that are trained with weightlifting pulling derivatives may provide a similar (PULL group) or greater (OL group) benefit. This is supported by previous literature that has shown that the magnitude of work performed during the load acceptance phase of weightlifting pulling derivatives may be similar [58] or greater [58,59,60] than that done during certain catching derivatives. As noted above, the OL group produced greater relative force during 11% of the total movement time compared to the CATCH group. Because the focus of the current study was on the propulsion phase of the SJ and CMJ, it is suggested that future research should compare the braking phase adaptations following training with weightlifting catching and pulling derivatives. Specifically, researchers may investigate variables such as braking mean force, time, peak force, and rate of force development.

Using percentages of the participants’ pre-intervention 1RM PC to program the pulling derivatives may be viewed as a limitation to the current study. If athletes use the PC regularly in their training programs, this may not be issue; however, alternative methods of prescribing pulling derivatives are required. Although a recent study examined loading effects with the jump shrug using percentages of body mass [36], it is clear that further research is needed to identify different loading methods. Although the current training program allowed participants to complete strength–endurance, strength, overreach, and taper phases, a second limitation may have been the length of the training program. The program was limited in part due to the length of the academic semester and the need to work around breaks within the academic calendar. This in turn prevented the prescription of a training phase dedicated to low-volume, high-load strength adaptations. Given the importance of relative muscular strength to athletic performance [61], and its potential impact on adaptations with weightlifting training [62], future studies may consider including such a phase in subsequent research.

## 5. Conclusions

The results of the current study showed that training with weightlifting pulling derivatives may produce greater SJ and CMJ adaptations compared to those obtained with catching derivatives. Specifically, training with a force and velocity overload stimulus with weightlifting pulling derivatives may enhance SJ height by increasing mean propulsion forces and decreasing propulsion time. However, the greatest improvements in CMJ performance may be realized by using submaximally loaded pulling derivatives. While further research is needed, practitioners are encouraged to use a combination of heavy and light loads when prescribing weightlifting pulling derivatives to enhance vertical jump performance.

## Figures and Tables

**Table 1:** Participant demographics for each training group.

	CATCH (*n* = 8)	PULL (*n* = 9)	OL (*n* = 8)
Age (years)	22.1 ± 3.3	22.2 ± 2.3	22.5 ± 1.2
Body mass (kg)	85.7 ± 14.3	84.3 ± 17.3	84.9 ± 13.2
Height (cm)	180.7 ± 6.1	179.6 ± 3.7	173.8 ± 9.9
Power clean experience (years)	6.4 ± 3.1	6.4 ± 2.4	6.6 ± 1.8
Relative 1RM power clean (kg · kg^−1^)	1.16 ± 0.10	1.19 ± 0.18	1.25 ± 0.16
Relative 1RM back squat (kg · kg^−1^)	1.64 ± 0.22	1.73 ± 0.17	1.76 ± 0.34

Notes: CATCH = catch group; PULL = pull group; OL = overload group; 1RM = one repetition maximum. Relative 1RM back squat strength was estimated using the participants’ heaviest loads lifted, sets, and repetitions of their most recent training phase completed prior to the study.

**Table 2:** Ten week resistance training program including sets × repetitions.

Training Block	Day 1	Day 2	Day 3
Strength–endurance, Weeks 1–3, 3 × 10	Back squat, Military press, Split squat, Bench press	Power clean from floor/*Clean pull from floor* Stiff-legged deadlift Bent-over row Pull-up	Power clean from floor/*Clean pull from floor* Back squat Incline bench press Bent-over row
Max-strength, Weeks 4–7, 3 × 5 + Overreach, Week 8, 5 × 5	Push press, Back squat, Bench press, Lunge	Mid-thigh power clean/*Mid-thigh pull* Power clean from floor/*Clean pull from floor* Stiff-legged deadlift Pull-up	Mid-thigh power clean/*Mid-thigh pull* Back squat Incline bench press Dumbbell row
Speed–strength, Weeks 9–10, 3 × 3, 3 × 2	Jerk ¼ squat + Squat jump, Bench press	CM power clean/*CM shrug* Hang power clean/*Hang high pull*	Jerk Hang power clean/*Jump shrug*

Notes: *Italics exercise* = weightlifting pulling derivative prescribed for Pull and Overload groups; CM = countermovement. The ¼ squats were performed using a concentric-only motion from squat rack safety bars (115°–125° knee angle), and squat jumps were performed from a knee angle of approximately 90°.

**Table 3:** Weekly relative intensity (set-repetition best) for traditional exercises and weightlifting derivative load (1RM power clean (PC) percent) progressions for the CATCH, PULL, and OL groups.

Wk	S × R	Day 1	Day 2	Day 3	CATCH	PULL	OL
		**Traditional Exercises**			**PC from Floor**	**CP from Floor**	**CP from Floor**
1	3 × 10	80%	80%	70%	55–57.5%	55–57.5%	75–77.5%
2	3 × 10	85%	85%	75%	57.5–60%	57.5–60%	77.5–80%
3	3 × 10	90%	90%	80%	60–62.5%	60–62.5%	80–82.5%
					**Mid-Thigh PC, PC from Floor**	**Mid-Thigh Pull, CP from Floor**	**Mid-Thigh Pull, CP from Floor**
4	3 × 5	85%	85%	70%	55–60%, 70–75%	55–60%, 70–75%	110–120%, 90–95%
5	3 × 5	90%	90%	75%	60–65%, 75–80%	60–65%, 75–80%	120–127.5%, 95–100%
6	3 × 5	95%	95%	77.5%	65–70%, 80–82.5%	65–70%, 80–82.5%	127.5–135%, 100–102.5%
7	3 × 5	80%	80%	65%	50–55%, 65–70%	50–55%, 65–70%	112.5–120%, 85–87.5%
8	5 × 5	85%	85%	75%	53–58%, 63–68%	53–58%, 63–68%	107–112%, 80–85%
					**CM PC, Hang PC**	**CM Shrug, Hang High Pull, Jump Shrug**	**CM Shrug, Hang High Pull, Jump Shrug**
9	3 × 3	90%	90%	77.5%	60–65%, 70–75% (Day 2) and 55–60% (Day 3)	60–65%, 70–75%, 55–60%	105–110%, 40–45%, 35–40%
10	3 × 2	85%	85%	75%	55–60%, 65–70% (Day 2) and 50–55% (Day 3)	55–60%, 65–70%, 50–55%	100–105%, 35–44%, 30–35%

Notes: Wk = week; S × R = sets × repetitions; Traditional exercises = non-weightlifting movements; CP = clean pull; CM = countermovement. Relative intensity may be interpreted as very light (65–70%), light (70–75%), moderately light (75–80%), moderate (80–85%), moderately heavy (85–90%), heavy (90–95%), and very heavy (100%) [45].

**Table 4:** Reliability statistics for squat jump (SJ) and countermovement jump (CMJ) variables.

Variable	Pre		MT1		MT2		Post	
	ICC (CI)	CV%	ICC (CI)	CV%	ICC (CI)	CV%	ICC (CI)	CV%
SJ Height	0.96 (0.91–0.98)	5.2	0.94 (0.85–0.97)	5.8	0.95 (0.89–0.98)	5.2	0.92 (0.83–0.97)	7.1
SJ MF	0.98 (0.96–0.99)	3.2	0.98 (0.97–0.99)	2.9	0.98 (0.95–0.99)	3.3	0.97 (0.93–0.99)	3.6
SJ Time	0.83 (0.62–0.93)	5.9	0.89 (0.75–0.95)	4.2	0.90 0.77–0.96)	3.7	0.77 (0.47–0.90)	5.8
CMJ Height	0.98 (0.95–0.99)	4.6	0.96 (0.92–0.98)	4.6	0.96 (0.90–0.98)	4.3	0.95 (0.88–0.98)	4.8
CMJ MF	0.99 (0.98–0.99)	2.4	0.99 (0.98–0.99)	1.8	0.99 (0.98–0.99)	1.9	0.99 (0.98–0.99)	2.0
CMJ Time	0.94 (0.86–0.97)	4.3	0.96 (0.91–0.98)	3.7	0.97 (0.92–0.98)	3.3	0.98 (0.94–0.99)	3.4

Notes: Pre = pre-intervention; MT1 = mid-test 1 (after 3 weeks); MT2 = mid-test 2 (after 7 weeks); Post post-intervention; ICC = intraclass correlation coefficient; CI = 95% confidence intervals; CV% = typical error expressed as a coefficient of variation percentage; MF = mean force.

**Table 5:** Descriptive squat jump (SJ) and countermovement jump (CMJ) from each testing session.

Variable		CATCH							PULL						OL						
		Pre	MT1			MT2		Post	Pre	MT1		MT2		Post	Pre	MT1		MT2		Post	
SJ Height (cm)	M	33.5	32.7			32.9		33.2	35.0	36.2		36.3		35.8	34.2	37.1		35.7		36.8	
	SD	4.8	3.8			3.1		4.5	6.8	4.7		6.5		6.2	6.9	7.3		7.0		6.9	
	*g*	−0.18		0.06			0.08		0.19		0.03		−0.08		0.40		−0.20		0.15		
		−**0.06**							**0.11**						**0.35**						
SJ MF (N·kg^−1^)	M	16.7	16.5			16.8		16.5	16.3	17.3		17.1		16.7	16.8	16.8		17.8		17.2	
	SD	1.0	1.0			1.3		0.9	0.8	0.7		0.8		0.8	2.0	1.5		1.7		1.2	
	*g*	−0.19			0.28		−0.23		1.29		−0.30		−0.48		0.02		0.57		−0.35		
		−**0.13**							**0.45**						**0.26**						
SJ Time (ms)	M	401	418 *			384		398	423	393		387		400 *	400	391		364		366	
	SD	37	46			37		34	40	24		27		25	66	33		25		27	
	*g*	0.38			−0.77		0.37		−0.85		−0.25		0.48		−0.18		−0.87		0.09		
		−**0.08**							−**0.65**						−**0.64**						
CMJ Height (cm)	M	34.4	33.7			34.0		35.6	35.1	36.3		36.4		38.4	35.5	37.7		37.1		38.1	
	SD	7.3	5.5			4.8		3.8	5.6	5.2		5.1		5.3	7.5	6.2		5.0		6.0	
	*g*	−0.10			0.04		0.36		0.22		0.00		0.37		0.30		−0.11		0.16		
		**0.19**							**0.57**						**0.35**						
CMJ MF (N·kg^−1^)	M	19.8	19.6			19.4		19.6	20.3	20.7		20.6		21.1	20.6	20.4		20.7		20.8	
	SD	1.5	1.1			1.3		1.0	1.8	2.1		2.0		2.5	1.0	2.2		1.2		1.5	
	*g*	−0.17			−0.13		0.18		0.17		0.03		0.18		−0.13		0.13		0.10		
		−**0.14**							**0.31**						**0.11**						
CMJ Time (ms)	M	258	263			264		260	249	247		247		244	235	249		243		243	
	SD	27	22			29		23	37	37		39		42	18	36		22		37	
	*g*	0.18			0.06		−0.15		−0.04		0.00		−0.08		0.47		−0.19		−0.01		
		**0.07**							−**0.12**						**0.25**						

*Notes:* Pre = pre-intervention; MT1 = mid-test 1; MT2 = mid-test 2; Post = post-intervention; MF = mean force; M = mean; SD = standard deviation; * = statistically greater than MT2 (*p* < 0.03); *g* = Hedge’s *g* effect sizes. Effect sizes display the changes between each testing session, while the bold *g* magnitude displays the changes from pre- to post-intervention.

## Supplementary Materials

The following are available online at https://www.mdpi.com/2411-5142/5/2/28/s1.
